# Vanillin reduction in the biosynthetic pathway of capsiate, a non-pungent component of *Capsicum* fruits, is catalyzed by cinnamyl alcohol dehydrogenase

**DOI:** 10.1038/s41598-022-16150-1

**Published:** 2022-07-20

**Authors:** Kaori Sano, Yuya Uzawa, Itsuki Kaneshima, Saika Nakasato, Masashi Hashimoto, Yoshiyuki Tanaka, Sachie Nakatani, Kenji Kobata

**Affiliations:** 1 Department of Chemistry, Faculty of Science, Josai University, 1-1, Keyakidai, Sakado, Saitama Japan; 2 Graduate School of Pharmaceutical Sciences, Josai University, 1-1, Keyakidai, Sakado, Saitama Japan; 3 Graduate School of Agriculture, Kyoto University, Kyoto, Japan

**Keywords:** Molecular biology, Plant molecular biology, Secondary metabolism, Enzyme mechanisms, Enzymes, Expression systems

## Abstract

*Capsicum* fruits synthesize capsaicin from vanillylamine, which is produced from vanillin in a reaction catalyzed by a putative aminotransferase (pAMT). Capsiate, a non-pungent compound that is structurally similar to capsaicin, is synthesized from vanillyl alcohol rather than vanillylamine. Vanillyl alcohol is possibly generated by the enzymatic reduction of vanillin, but the enzyme responsible for this reaction is unknown. In the present study, we revealed that the vanillin reductase in the capsiate biosynthetic pathway is cinnamyl alcohol dehydrogenase (CAD), which is an enzyme involved in lignin synthesis. The reduction of vanillin to vanillyl alcohol was greater in the mature red fruit placental extract than in the immature green fruit placental extract. This reduction was suppressed by both *N*-(*O*-hydroxyphenyl) sulfinamoyltertiobutyl acetate, a specific inhibitor of CAD, and ethylenediaminetetraacetic acid, a metalloenzyme inhibitor. The *CaCAD1* transcript levels in the placenta were higher in the red fruits than in the green fruits. A recombinant CaCAD1 protein obtained using an *Escherichia coli* expression system reduced vanillin to vanillyl alcohol. This reaction was suppressed by the CAD inhibitors. These results strongly suggest that CAD is the enzyme that catalyzes the reduction of vanillin to vanillyl alcohol during capsiate biosynthesis. Syntenic analyses indicated that genes encoding CAD and capsaicin synthase (*Pun1*) involved in capsiate biosynthesis were acquired before the *pAMT* gene during the evolution of the family Solanaceae. This raises the possibility that in the genus *Capsicum*, the capsiate biosynthetic pathway emerged before the pAMT-encoding gene was acquired as the final trigger for capsaicin biosynthesis.

## Introduction

Chili pepper (*Capsicum*), a member of the family Solanaceae, produces fruits that contain capsaicinoids, which are unique pungent alkaloids. Capsaicinoids can repel herbivorous mammals, but because birds lack capsaicinoid receptors and do not perceive pungency, they are able to serve as chili pepper seed dispersers^1^. The pungency of capsaicinoids has made chili pepper one of the most economically important spice crops. About 40 million tons of chili pepper fruits are produced and consumed worldwide per year. In particular, capsaicin, the major capsaicinoid, has attracted the attention of researchers because of its diverse bioactivities that make it potentially useful as an analgesic and for treating cancer, inflammation, oxidative stress, and obesity^2^.

**Figure 1:**
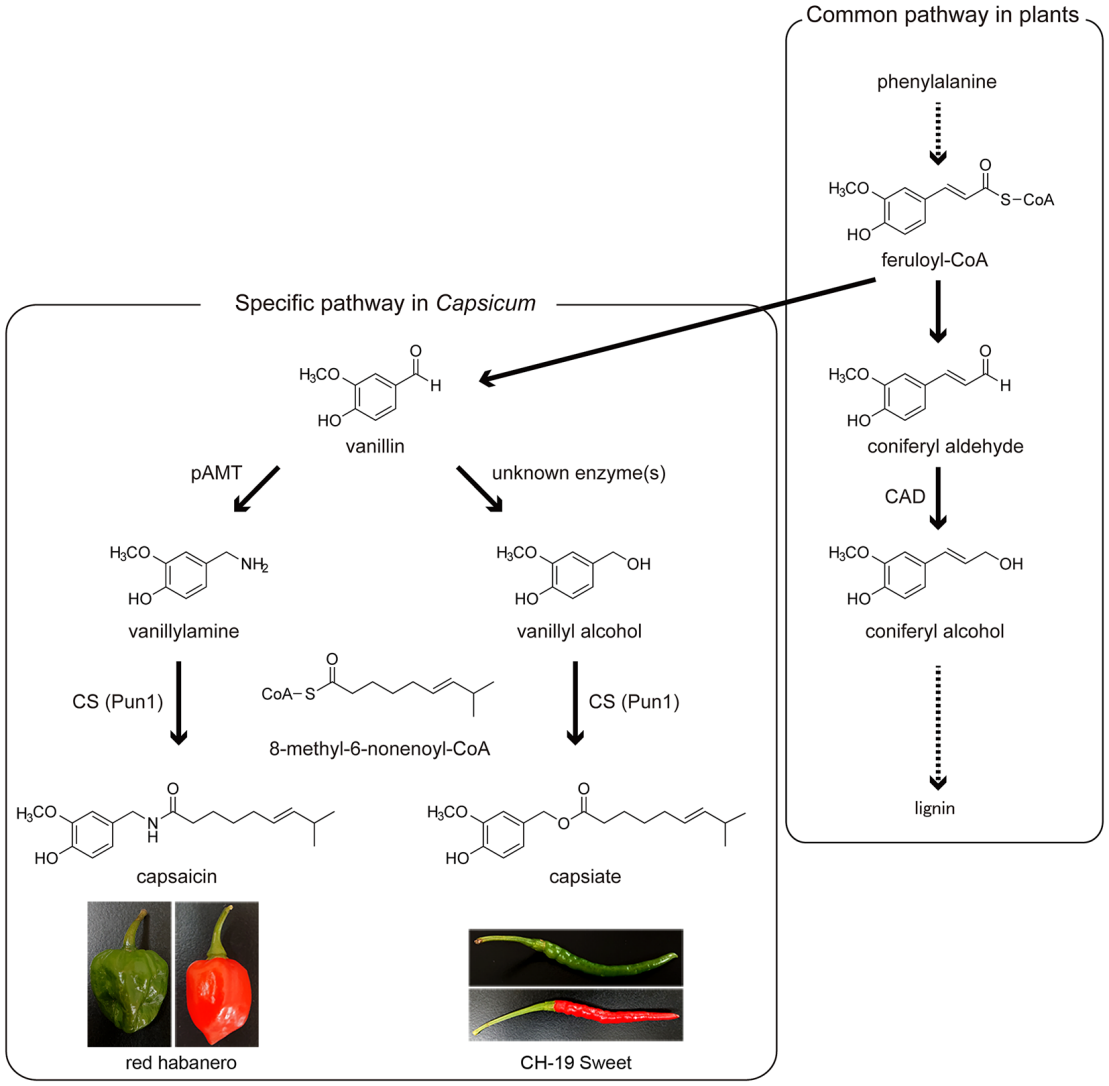
Partial capsaicin, capsiate, and lignin biosynthetic pathways. *Capsicum*-specific capsaicin and capsiate biosynthetic pathways are branched from the lignin biosynthetic pathway in plants. The intermediate products and the main enzymes catalyzing each reaction are shown. Examples of varieties producing capsaicin and capsiate are provided at the bottom.

The biosynthetic pathway of capsaicin is well characterized. Capsaicin is synthesized by the enzymatic condensation of vanillylamine and a branched fatty acid. The condensation is catalyzed by the capsaicin synthase encoded by *Pun1*^3^. During the biosynthesis of capsaicin, vanillylamine is produced from phenylalanine via the phenylpropanoid pathway, with putative aminotransferase (pAMT) likely essential for converting vanillin to vanillylamine (Fig. 1)^4,5^. In 1989, the *Capsicum annuum* variety CH-19 Sweet was obtained as a non-pungent mutant of its spicy parent, CH-19, originating from Thailand. This was the first variety with a non-functional pAMT^6^. Further analyses showed that the *pAMT* gene of CH-19 Sweet had a 1-bp insertion in the 16th exon, which results in the deletion of 29 amino acid residues at the C-terminal of the functional pAMT protein^7^. A Japanese low-pungency landrace, Himo (*C. annuum*), also has a non-functional pAMT^8^, and its loss of function is due to the substitution of one amino acid residue. Interestingly, these varieties with non-functional pAMTs accumulate capsinoids instead of capsaicinoids^9,10^. Capsinoids are structurally similar to capsaicinoids, but they have an ester bond instead of an amide bond between the aromatic ring and the branched fatty acid. Because of this difference, capsinoids have almost no pungency, unlike capsaicinoids. However, capsiate, the major capsinoid, still has diverse capsaicin-like bioactivities, including an anti-obesity effect that makes it suitable for use in weight-loss supplements^2,11^.

The proposed biosynthetic pathway of capsiate from vanillin is as follows: vanillin is reduced to vanillyl alcohol by an unknown enzyme(s) and then vanillyl alcohol and a branched fatty acid are dehydrated and condensed by the action of capsaicin synthase (Fig. 1)^12^. Although the existence of capsiate is already well known, the key enzyme that reduces vanillin in the capsiate biosynthetic pathway has not been identified. The identification of this enzyme may lead to enhanced commercial production of capsiate and/or capsaicin. It may also shed light on how *Capsicum* acquired the ability to synthesize capsaicinoids and capsinoids during evolution. It has been reported that the antisense oligonucleotide suppression of *pAMT* in a pungent *Capsicum* strain leads to a decreased capsaicin content and the significant accumulation of capsiate^7^. This is similar to what occurs in the non-functional pAMT mutants CH-19 Sweet and Himo. This suggests that capsiate can be produced in varieties that predominantly synthesize capsaicin. Therefore, in this study, we focused on the possibility that an enzyme(s) in *Capsicum* species might reduce vanillin to vanillyl alcohol in the absence of a functional pAMT, thereby constituting the capsiate biosynthetic pathway.

We hypothesized that cinnamyl alcohol dehydrogenase (CAD), which is an essential enzyme in the biosynthetic pathway of lignin^13^, a major cell wall component, may be the enzyme responsible for the reduction of vanillin for the following reasons. First, vanillin is structurally similar to coniferyl aldehyde, which is reduced to coniferyl alcohol by CAD in the lignin biosynthetic pathway (Fig. 1). Second, CAD belongs to a metalloenzyme family whose members catalyze the reduction of various phenylpropenyl aldehyde derivatives to their corresponding alcohols, using NADPH as a proton donor^14^. Third, because vanillin is a by-product of lignin biosynthesis, an enzyme in the lignin biosynthetic pathway, such as CAD, may catalyze reactions involving vanillin (Fig. 1)^15^. In the present study, we focused on CAD to verify its vanillin reductase activity and clarify the capsiate biosynthetic pathway. We also investigated the evolutionary process by which *Capsicum* acquired the capsaicin synthesis system by analyzing the synteny among related genes.

## Results

### Vanillin reduction activity in the placental extract of CH-19 sweet

**Figure 2:**
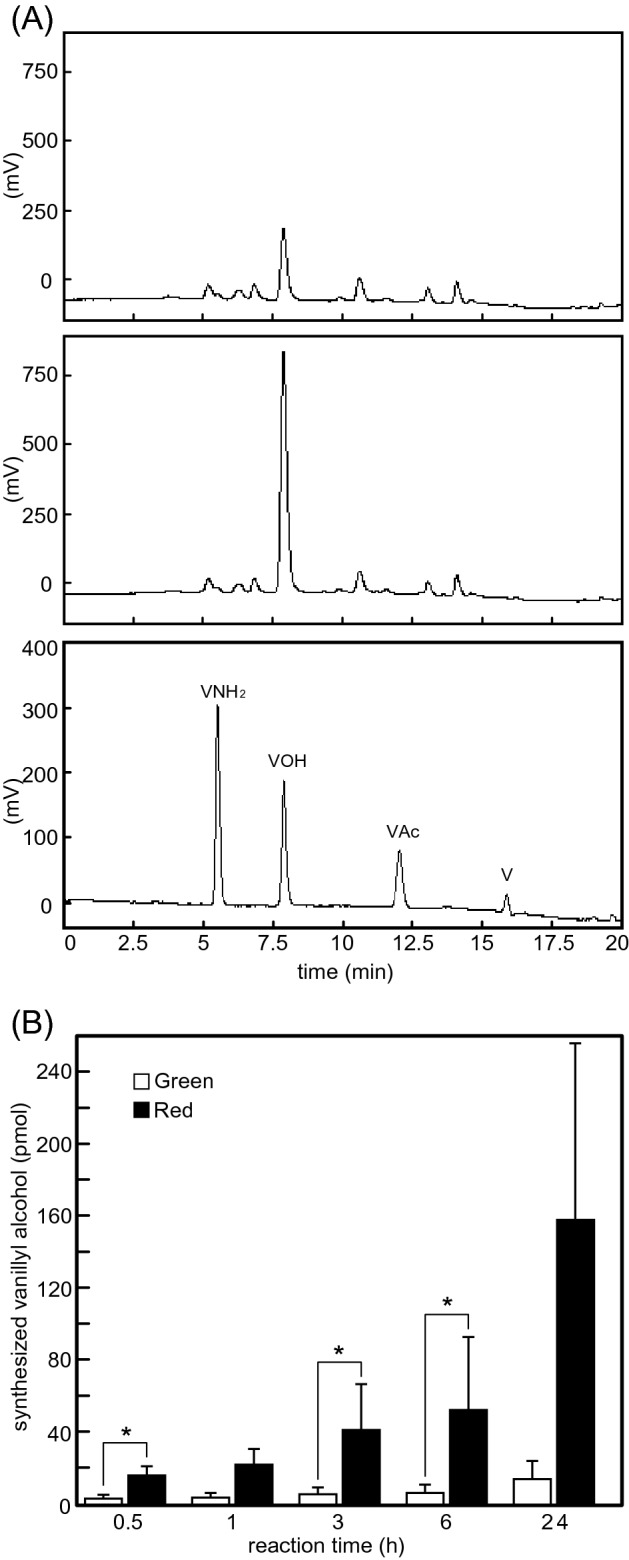
Vanillin reduction activity of CH-19 Sweet placental extracts. (**A**) Typical chromatograms for the HPLC analysis of vanillyl alcohol production in a green fruit placental extract (fluorescence detection: 280 nm excitation wavelength and 320 nm emission wavelength). Vanillyl alcohol contents before the reaction (top) and after a 60-min reaction (middle) are presented. Vanillin derivatives (V: vanillin; VOH: vanillyl alcohol; VNH_2_: vanillylamine; and Vac: vanillic acid) were separated by HPLC as controls (bottom). (**B**) Amount of vanillyl alcohol synthesized by placental extracts at each reaction time-point. White and black bars indicate the results for the green fruits (*n* = 7) and red fruits (*n* = 5), respectively. Asterisks indicate a significant difference (*P* < 0.05) as determined by Welch’s *t*-test.

The vanillin reduction activity in extracts obtained from the placenta was compared between immature green and mature red fruits of CH-19 Sweet. Because the extracts originally contained vanillyl alcohol, the production of vanillyl alcohol from externally added vanillin was determined as the vanillin reduction activity (Fig. 2A). Figure 2B presents the vanillin reduction activity of the placental extracts. Although the vanillin reduction activity was detected in both green and red fruit placental extracts, it was higher in the red fruit extract than in the green fruit extract at all examined reaction time-points. After 24 h, the amount of synthesized vanillyl alcohol was about 10-times greater in the red fruit extract than in the green fruit extract. When a boiled extract was reacted with vanillin, no vanillyl alcohol was synthesized (Table S1), suggesting that vanillyl alcohol was synthesized by the enzyme(s) contained in the extract.

### Inhibition of the vanillin reduction activity of CH-19 sweet

**Figure 3:**
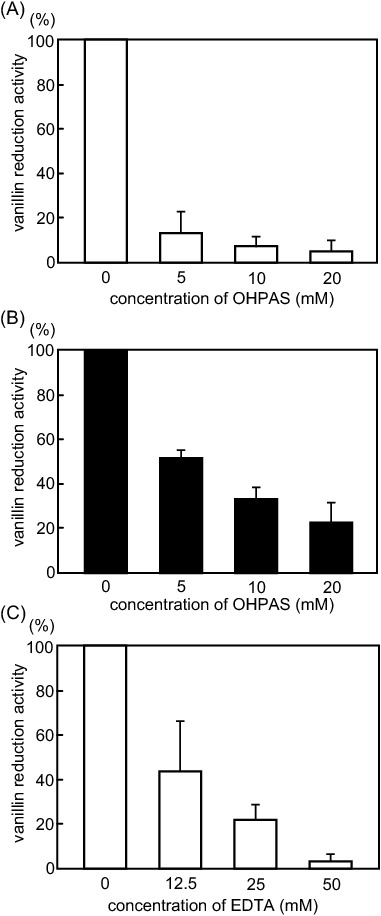
Inhibition of the vanillin reduction activity of CH-19 Sweet placental extracts by OHPAS and EDTA. Vanillin reduction activity of placental extracts from (**A**) immature green and (**B**) mature red fruits was inhibited by 0–20 mM OHPAS. (**C**) The activity of immature green fruits was inhibited by 0–50 mM EDTA. The amount of vanillyl alcohol synthesized without inhibitors was set as 100% and used to calculate the degree of inhibition. Experiments were performed using three individual fruits (*n* = 3).

Next, we investigated the inhibitors of vanillyl alcohol production (i.e., vanillin reduction activity) in the placental extract. First, we examined the effects of *N*-(*O*-hydroxyphenyl) sulfinamoyltertiobutyl acetate (OHPAS), which is a specific inhibitor of poplar CAD^16^. The addition of OHPAS to the reaction mixtures containing the placental extracts of green and red fruits and vanillin decreased the amount of vanillyl alcohol produced in a dose-dependent manner (Fig. 3A, B). In the green fruit extract, 5 mM OHPAS inhibited the reduction of vanillin by about 90%, whereas in the red fruit extract, 20 mM OHPAS inhibited the activity by about 80%. However, OHPAS also suppressed the pAMT-catalyzed synthesis of vanillylamine from vanillin in the placental extracts of another *Capsicum* species (red habanero (Fig. S1A). Therefore, we experimented with another inhibitor to inhibit the activity of CAD.

Ethylenediaminetetraacetic acid (EDTA) was used as another inhibitor. Considering CAD is a metalloenzyme with a zinc ion in its active site, we thought that if CAD catalyzes the synthesis of vanillyl alcohol, it may be inhibited by EDTA. The vanillyl alcohol synthesis reaction involving the green fruit placental extract was inhibited by EDTA in a concentration-dependent manner (Fig. 3C). Because EDTA did not affect the pAMT-catalyzed synthesis of vanillylamine from vanillin in the placental extracts of red habanero, the EDTA concentration used in this experiment was considered to be appropriate (Fig. S1). These results suggested that CAD is the enzyme catalyzing the reduction of vanillin to generate vanillyl alcohol in the placenta.

### Quantification of *CAD, pAMT*, and *Pun1* transcripts

**Figure 4:**
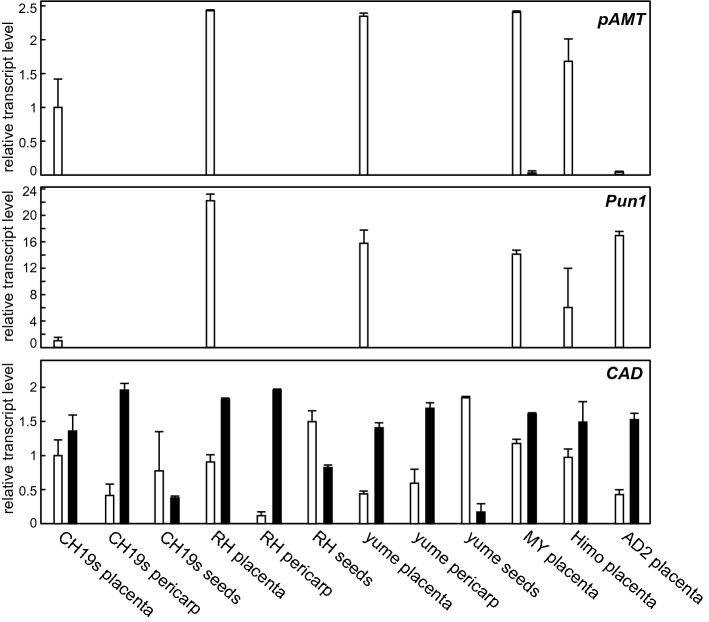
Quantitative real-time PCR analyses of *CAD, pAMT,* and *Pun1*. The transcript levels of *pAMT* (top), *Pun1* (middle), and *CAD* (bottom) in the placenta, pericarp, and seeds of CH-19 Sweet (CH19s), red habanero (RH), Yume-matsuri (yume), Moruga Yellow (MY), Himo, and Aji Dulce strain2 (AD2) were compared. Expression was normalized against that of average of three control genes. The relative transcript level of each gene was calculated according to the comparative Ct method, with the expression level of each gene in CH-19 Sweet set to 1. White and black bars indicate immature green and mature red fruits, respectively. Each experiment was performed using RNA extracted from three different individual fruits (*n* = 3).

We conducted quantitative real-time PCR (qPCR) analyses to compare the *CAD, pAMT*, and *Pun1* transcript levels in the placenta, pericarp, and seeds of the immature green and mature red fruits of CH-19 Sweet, red habanero, Yume-matsuri, Moruga Yellow, Himo, and Aji Dulce strain 2 (Fig. 4). Both *pAMT* and *Pun1*, which are capsaicin synthesis-related genes, were highly transcribed only in the placenta of green fruits; their transcription was barely detectable in the other examined tissues in all varieties. This result was consistent with the location and timing of capsaicin synthesis. In contrast, *CAD* was ubiquitously expressed in the green fruits (i.e., not only in the placenta, which is where capsiate is synthesized). Additionally, *CAD* tended to be more highly expressed in the red fruit placenta than in the green fruit placenta (Fig. S2), which is in accordance with the results of the vanillin reduction activity analysis using placental extracts (Fig. 2B).

### Preparation and characterization of recombinant CaCAD1

The full-length *CAD* cDNA was cloned from mRNA extracted from CH-19 Sweet and red habanero placentas. The amino acid sequences deduced from the *CAD* cDNA sequences were 100% identical between CH-19 Sweet and red habanero (Fig. S3). Moreover, all of the conserved amino acid residues in the active site of CADs in other plants were conserved in the CH-19 Sweet and red habanero CADs (Fig. S3). These genes were identical to *CaCAD1* (Accession no: NM001324580)^17^. The amino acid sequence of the CH-19 Sweet CAD matched that of CaCAD1.

Recombinant CaCAD1 (rCaCAD1) was obtained using the *Escherichia coli* expression system and purified by Ni–NTA column chromatography. In an SDS-PAGE analysis, a single band (approximately 39.6 kDa) matched the molecular weight estimated from the cDNA (Fig. S4). A total of 12.25 mg rCaCAD1 protein was obtained from a 250-mL culture system.

**Figure 5:**
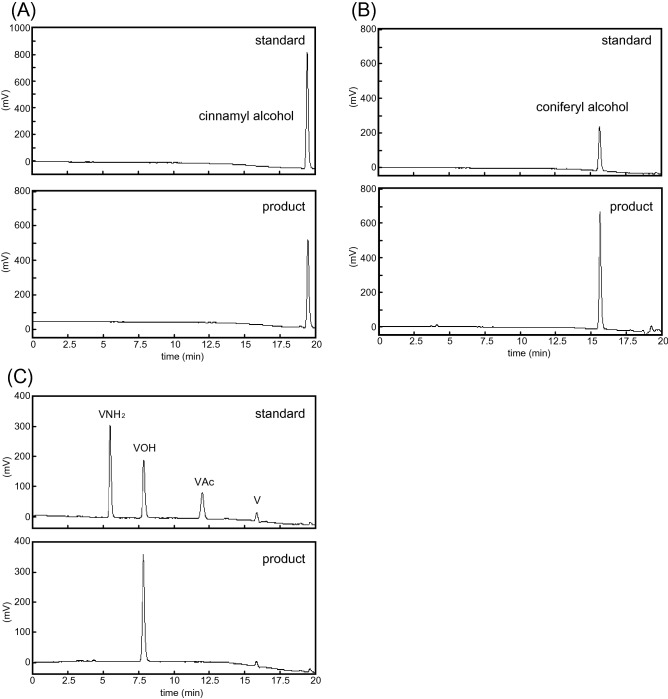
HPLC chromatograms of the products of reactions between rCaCAD1 and several substrates. Chromatograms for the products of the reactions between rCaCAD1 and (**A**) cinnamaldehyde, (**B**) coniferyl aldehyde, and (**C**) vanillin are presented. The components in the reaction mixture were analyzed by HPLC (fluorescence detection: 280 nm excitation wavelength and 320 nm emission wavelength). The HPLC chromatograms for the following standards are provided (top): (**A**) cinnamyl alcohol, (**B**) coniferyl alcohol, and (**C**) vanillin derivatives (V: vanillin; VOH: vanillyl alcohol; VNH_2_: vanillylamine; and VAc: vanillic acid). After a 30-min reaction, the products were analyzed by HPLC (bottom). Cinnamaldehyde, coniferyl aldehyde, and vanillin were hardly detectable at this wavelength, which explains the lack of peaks for the unreacted substrates.

The reaction between 5 µg rCaCAD1 and cinnamaldehyde, a typical substrate of CAD, resulted in the synthesis of 25.1 nmol cinnamyl alcohol in 30 min (Fig. 5A). Furthermore, coniferyl aldehyde, which is usually reduced by CAD in the lignin biosynthetic pathway, was converted to 10.6 nmol coniferyl alcohol by 5 µg rCaCAD1 (Fig. 5B). These results indicate that functional rCaCAD1 was generated by the *E. coli* expression system.

When 5 µg rCaCAD1 was reacted with vanillin, 6.4 nmol vanillyl alcohol was synthesized in 30 min (Fig. 5C). The reaction involving boiled rCaCAD1 and vanillin did not result in the synthesis of vanillyl alcohol (Table S2). The absence of the proton donor NADPH also resulted in a lack of vanillyl alcohol production (Table S2). These results confirmed that CaCAD1 can catalyze the reduction of vanillin to generate vanillyl alcohol.

### Inhibition of rCaCAD1 activity by OHPAS and EDTA

**Figure 6:**
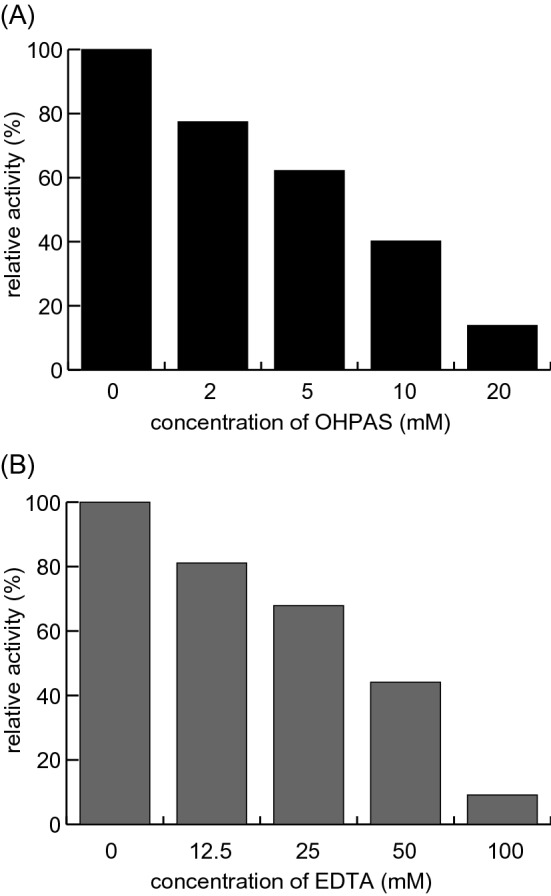
Inhibition of the vanillin reduction activity of rCaCAD1 by OHPAS and EDTA. Inhibition of the vanillin reduction activity of rCaCAD1 by 0–20 mM OHPAS (**A**) and 0–100 mM EDTA (**B**). The amount of vanillyl alcohol synthesized without inhibitors was set as 100% and used to calculate the degree of inhibition.

A previous study revealed that OHPAS is a specific inhibitor of poplar CAD^16^. The poplar CAD and CaCAD1 full-length amino acid sequences were determined to be 81% similar (Fig. S3). In our experimental conditions, OHPAS suppressed the vanillin reduction activity of rCaCAD1 in a concentration-dependent manner. In reaction mixtures containing 10 µg rCaCAD1, 5 mM OHPAS suppressed the activity to 60%, whereas 20 mM OHPAS suppressed the activity to 20% (Fig. 6A). Additionally, when used as a metalloenzyme inhibitor, EDTA suppressed the vanillin reduction activity of rCaCAD1 in a concentration-dependent manner (Fig. 6B). These results confirmed that OHPAS and EDTA can efficiently inhibit *Capsicum* CAD.

### Syntenic analysis of *CAD*, *pAMT*, and *Pun1*

**Figure 7:**
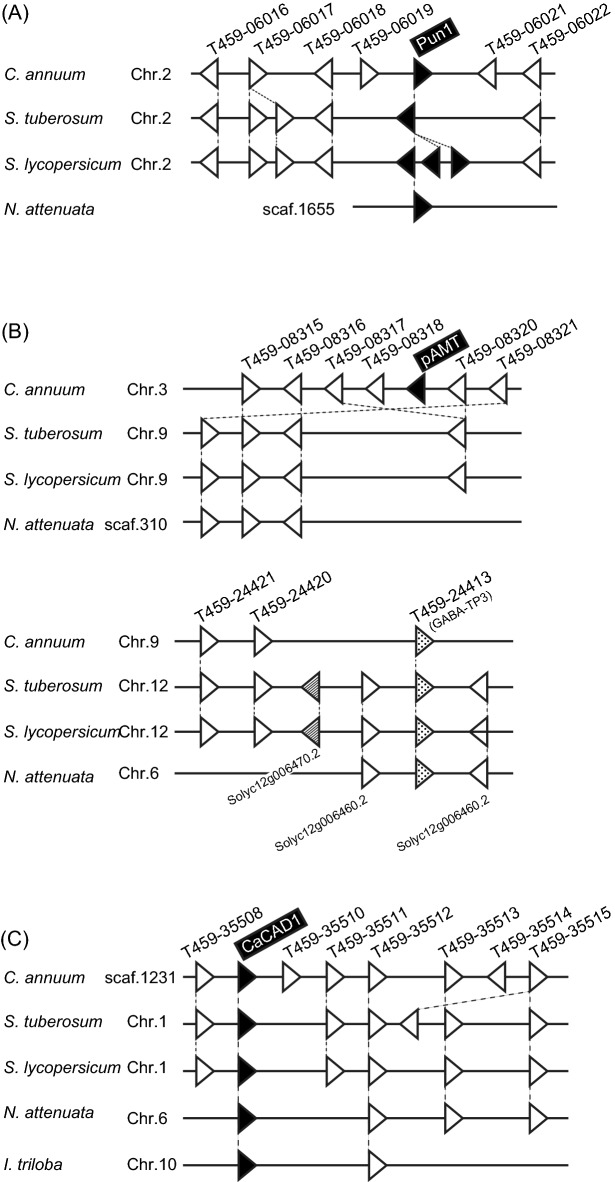
Syntenic analyses of *C. annuum, S. tuberosum*, *S. lycopersicum, N. attenuata*, and *I. triloba* genes*.* (**A**) Synteny around *Pun1*. Black triangles indicate *Pun1* and orthologous genes (*S. tuberosum*: PGSC0003DMG400007310; *S. lycopersicum*: Solyc02g081740.2, Solyc02g081745.2, and Solyc02g081760.2; *N. attenuata*: ASAT3_8). (**B**) Synteny around *pAMT* and homologous genes. The black triangle indicates *pAMT*, whereas dotted and diagonal patterned triangles indicate homologous genes. (**C**) Synteny around *CaCAD1*. Black triangles indicate *CaCAD1* and orthologous genes (*S. tuberosum*: PGSC0003DMG401025767; *S. lycopersicum*: Solyc01g107590; *N. attenuata*: CAD19; *I. triloba*: itb10g24120). Distances between intergenic regions and gene coding regions are arbitrary. Gene names on the top and bottom are from the *C. annuum* and *S. lycopersicum* genomes, respectively.

To estimate when the capsaicin and capsiate biosynthetic pathways were established during evolution, we analyzed the synteny of *CAD*, *pAMT*, and *Pun1* genes among the genomes of the Solanaceae plant species *C. annuum*, *Solanum tuberosum* (potato), *Solanum lycopersicum* (tomato), and *Nicotiana attenuata* (tobacco) as well as the genome of the Convolvulaceae species *Ipomoea triloba* (three-lobed morning glory) in the order Solanales. First, we performed a comparative genomic analysis of *Pun1*, which is essential for the final catalytic reaction in the capsaicin and capsiate biosynthetic pathways (Fig. 7A). The BLAST searches of the whole genome sequences of *C. annuum* and its relatives revealed that the *C. annuum*, *S. tuberosum*, and *N. attenuata* genomes include a single copy of *Pun1*, whereas the *S. lycopersicum* genome contains three tandem repeats of *Pun1* (black triangles in Fig. 7A). The genomic synteny of these *Pun1* genes was well conserved, indicating that *Pun1* was present in the common ancestor in the family Solanaceae^18,19^.

Next, we searched for *pAMT*, which is essential for the capsaicin biosynthetic pathway. The BLAST search of the *C. annuum* whole genome sequence identified three sequences that were similar to *pAMT*. The first sequence was revealed to be conserved among Solanaceae plants (dotted triangles in Fig. 7B) and duplicated in *Solanum* species (diagonal patterned triangles in Fig. 7B). Regarding the second sequence, there was no conserved synteny among Solanaceae plants. The third sequence was identified as *pAMT*, which was specific to *C. annuum*, although genomic synteny was conserved (black triangle in Fig. 7B). These results indicate that *pAMT*, which is essential for the synthesis of the pungency-related compound capsaicin, was specifically acquired by the genus *Capsicum*.

Finally, we searched for *CaCAD1*, which encodes the enzyme that catalyzes the generation of vanillyl alcohol. The results of the BLAST search and genome synteny analysis revealed that *CaCAD1* is highly conserved in the genomes of *C. annuum* and its relatives as a single-copy gene (Fig. 7C). These findings suggest that the capsiate biosynthetic pathway may have already been evolutionarily established before pAMT was acquired by species in the genus *Capsicum* during evolution.

## Discussion

### CAD catalyzes the reduction of vanillin to vanillyl alcohol in the capsiate biosynthetic pathway

Capsaicin, an important pungent compound, is a secondary metabolite uniquely synthesized in *Capsicum* species; its biosynthetic pathway and related genes have been well studied. Both *pAMT* and *Pun1*, which encode enzymes involved in the synthesis of capsaicin from vanillin, are highly expressed in the placenta of all pungent *Capsicum* species studied to date. Therefore, loss-of-function mutations in *Pun1* or *pAMT* result in a loss of pungency. In fact, non-pungent green pepper (pimento) and paprika have dysfunctional *Pun1* genes^20,21^. Mutants with a non-functional pAMT also produce non-pungent fruit, and some of them, such as CH-19 Sweet and Himo, accumulate capsiate instead of capsaicin in the fruit placenta^7,8^.

Capsiate was first identified as a novel capsaicin-like substance in non-pungent *Capsicum* fruit^9,10^. The biosynthetic pathway of capsiate is similar to that of capsaicin, but the precursor for the synthesis of capsiate from vanillin is vanillyl alcohol instead of vanillylamine^22^. Analyses of non-pungent strains, such as CH-19 Sweet, demonstrated that a mutation in *pAMT* results in the accumulation of capsiate^7^. Notably, despite the loss of a functional pAMT, no residual vanillin was present in the mutants we tested (unpublished data). Therefore, we thought that another enzyme must metabolize vanillin, with the vanillyl alcohol produced following the reduction of vanillin used for capsiate biosynthesis. We hypothesized that CAD is involved in the reduction of vanillin to synthesize vanillyl alcohol.

The placental extracts of CH-19 Sweet reduced vanillin to vanillyl alcohol (Fig. 2). Capsiate is mainly synthesized in immature green fruits^23,24^. However, less vanillyl alcohol was synthesized by green fruit placental extracts than by red fruit placental extracts (Fig. 2B). A previous study on CAD in poplar indicated that this enzyme is specifically inhibited by OHPAS, which does not have any affinity for other plant zinc metalloenzymes or phenolic enzymes^16^. In the present study, OHPAS suppressed the vanillin reduction activity of the placental extracts (Fig. 3A, B) and rCaCAD1 (Fig. 6A), but it also inhibited the activity of pAMT, an enzyme that also metabolizes vanillin (Fig. S1A). Therefore, we used EDTA as another inhibitor. The addition of EDTA inhibited the vanillin reduction activity of the CH-19 Sweet placental extract and rCaCAD1, whereas it did not affect the vanillylamine synthesis activity of pAMT in the red habanero placental extract (Fig. 3A, B, S1B). These results confirm that pAMT is not involved in vanillin reduction. Our findings strongly suggest that CAD is the enzyme that produces vanillyl alcohol from vanillin in *Capsicum *in vivo. Thus, the entire pathway for the synthesis of capsiate from vanillin has been elucidated.

### CAD compensates for the functional loss of pAMT, converting vanillin to vanillyl alcohol in the placenta of *Capsicum* fruits

According to the qPCR analysis, *pAMT* and *Pun1* were transcribed in placentas of all examined *Capsicum* varieties (Fig. 4). High *pAMT* and *Pun1* transcript levels were detected in the immature green fruits, which was in contrast to the very low transcript levels in the mature red fruits. Because capsaicin is mainly synthesized in immature green fruits^23,24^, the timing of *pAMT* and *Pun1* transcript accumulation coincided with that of capsaicin synthesis. Capsiate is also mainly synthesized in immature green fruits^25^. This phenomenon suggests Pun1, which is a capsaicin synthase, is also involved in the final step of the capsiate biosynthetic pathway^12,26^. However, *CAD* was expressed in all examined tissues, with higher expression levels in the red fruit placenta than in the green fruit placenta (Figs. 4 and S2). Although we identified CAD as the enzyme that reduces vanillin to vanillyl alcohol, the direct precursor of capsiate, the location and timing of *CAD* expression was inconsistent with the location and timing of capsiate accumulation. In general, the primary function of CAD is its activity in lignin synthesis throughout the plant body. Thus, the expression pattern of CAD positively correlates with lignin accumulation^27^. Therefore, it is no wonder that *CAD* expresses independently with *pAMT* and *Pun1*. These results suggest that the CAD we focused on is not a specialized enzyme for vanillin reduction and that it metabolizes vanillin via an accompanying aldehyde reduction activity if pAMT is non-functional. In fact, the loss of a functional pAMT in some *Capsicum* species results in the accumulation of capsiate instead of capsaicin^7,8^. Furthermore, the silencing of pAMT-encoding genes also leads to the accumulation of capsiate rather than capsaicin^7^. This may explain the high *CAD* transcript levels in red fruits. According to the results so far, it seems that *Capsicum* species usually synthesize capsaicin in reactions catalyzed by pAMT and Pun1, but CAD can compensate for a non-functional pAMT to maintain vanillin metabolism, resulting in the reduction of vanillin followed by the accumulation of capsiate.

It was recently reported that the transcription factor MYB31 simultaneously upregulates the expression of several genes involved in capsaicin synthesis, including *pAMT* and *Pun1*^28,29^. The *CAD* expression pattern was unrelated to the MYB31-regulated expression of these genes. Accordingly, the original function of CAD was not associated with vanillin metabolism in the capsiate biosynthetic pathway.

### Syntenic analysis of structural genes in the capsaicin and capsiate biosynthetic pathways

**Figure 8:**
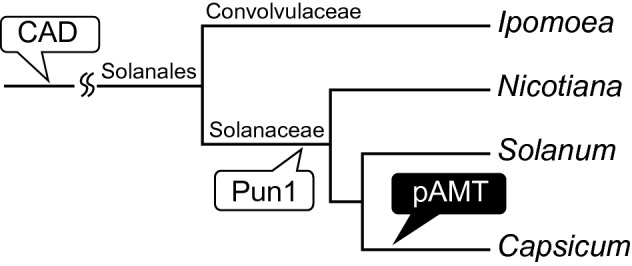
Schematic drawing of the evolutional process of gene acquisition. Simple phylogenetic relationship between Convolvulaceae and Solanaceae. The presented timing of the acquisition of *pAMT*, *CAD*, and *Pun1* was predicted on the basis of syntenic analyses. The branch length of the phylogenetic tree does not represent an evolutionary time scale.

Capsaicin biosynthesis is considered important for the coevolution of *Capsicum* and birds. *Capsicum* seeds retain their ability to germinate after digestion by birds, but not after digestion by mammals. The widespread dispersal of seeds by birds has expanded the habitat of *Capsicum* and mating with distant conspecifics has contributed to the genetic diversity of *Capsicum* species^30^. Because mammals have a receptor for capsaicin to perceive pungency, but birds do not, capsaicin biosynthesis may enhance *Capsicum* survival via the selective predation by birds^1^. How *Capsicum* acquired the mechanism underlying capsaicin biosynthesis during evolution is a topic of interest. Although the *C. annuum* genome contains several genes encoding aminotransferases, the functions of these enzymes apparently do not compensate for the function of pAMT. More specifically, a loss-of-function mutation in the pAMT-encoding gene results in a lack of capsaicin biosynthesis in several cultivars, including CH-19 Sweet^7^. Therefore, pAMT is the only aminotransferase that contributes to capsaicin biosynthesis. Interestingly, syntenic analyses showed that *pAMT* was acquired by species in the genus *Capsicum*, whereas *Pun1*, which encodes an enzyme that functions downstream of pAMT, was acquired in a common ancestor in the family Solanaceae^18,19^. Considering that CAD, which is involved in lignin synthesis, is present in most plants, it is possible that the capsiate biosynthetic pathway was established earlier than the capsaicin biosynthetic pathway during the evolution of *Capsicum* (Fig. 8). However, because it is unknown when *Capsicum* acquired the ability to synthesize vanillin, we cannot say for certain when the capsiate biosynthetic pathway was established. Additionally, because no pAMT orthologs have been detected in plants other than *Capsicum* species (Fig. 7B), the acquisition of pAMT and the establishment of the capsaicin biosynthetic pathway during *Capsicum* evolution may have been a major event underlying the success of the genus *Capsicum*. The mechanism mediating vanillin synthesis should be elucidated next.

## Methods

### Plant materials

*Capsicum annuum* CH-19 Sweet seeds were supplied by Ajinomoto Co., Inc. (Tokyo, Japan). The CH-19 Sweet, red habanero (*Capsicum chinense*), and Himo (*C. annuum*) plants were grown at the experimental farm of Josai University (Saitama, Japan), whereas Yume-matsuri (*C. annuum*), Moruga Yellow (*C. chinense*), and Aji Dulce strain 2 (*C. chinense*) plants were grown at the experimental farm of Kyoto University (Kyoto, Japan).

### Quantitative real-time PCR analyses

Total RNA was extracted from the placenta, pericarp, and seeds of green and red fruits of CH-19 Sweet, red habanero, Himo, Yume-matsuri, Aji Dulce strain 2, and Moruga Yellow using the Fast Gene RNA Premium kit (Nippon Genetics Co., Ltd., Tokyo, Japan) according to the manufacturer’s instructions. The extracted total RNA (0.5 µg) served as the template for the synthesis of cDNA using the PrimeScript RT Master Mix (Takara Bio Inc., Otsu, Japan). The *CAD, pAMT*, and *Pun1* transcript levels were determined by qPCR with the LightCycler 96 instrument (Roche Diagnostics, Penzberg, Germany). *β-actin* (AY572427), CaUBIQUITIN (DQ975458.1), and Elongation factor 1-alpha (AY496125) genes were used as an internal reference control. The specificity of each primer set (Table S3) was confirmed by a melting curve analysis. Each experiment was performed using RNA extracted from three different individual fruits (*n* = 3). The relative expression level of each gene was calculated using the comparative Ct method, with the expression level of each gene in CH-19 Sweet set to 1.

### Preparation of placental extracts

*Capsicum* fruits at different developmental stages (immature green fruits and mature red fruits) were dissected to isolate the placenta. Next, 100 mg placenta were homogenized in 1 mL 20 mM potassium phosphate buffer (PKB), pH 6.8. After a centrifugation at 18,000 g at 4 °C for 5 min, the supernatant was collected and filtered using a syringe filter (pore size 0.45 µm). The filtrate was dialyzed against 20 mM phosphate-buffered saline (PBS), pH 7.2, at 4 °C for 2 h and then used as the placental extract for enzymatic analyses.

### Cloning of the *CAD* cDNA from CH-19 sweet and red habanero

Total RNA was extracted as described above and then cDNA was synthesized from 0.5 µg total RNA using the PrimeScript 1st strand cDNA Synthesis Kit (Takara Bio Inc.). Each full-length cDNA fragment was amplified using the primers listed in Table S4. The PCR fragments were inserted into the T-vector pMD20 (Takara Bio Inc.) and then sequenced.

### Generation of recombinant CaCAD1

The full-length cDNA of *CaCAD1* with a His tag was transferred into the expression vector pET3c (Promega, Madison, WI, USA). *Escherichia coli* strain BL21 Star (DE3) cells (Thermo Fisher Scientific, Waltham, MA, USA) were transformed with the *CaCAD1/pET3c* recombinant plasmid. The BL21 Star (DE3) cells harboring *CaCAD1/pET3c* were then grown in 20 mL LB culture medium containing 50 µg/mL carbenicillin at 37 °C in a shaking incubator for 4 h. The culture was added to 250 mL prewarmed LB culture medium containing carbenicillin and then incubated at 37 °C with shaking. When the absorbance at 600 nm reached 0.6, isopropyl β-D-thiogalactoside was added for a final concentration of 1 mM. After a 4-h incubation with shaking, cells were harvested by a centrifugation at 3200 g for 10 min. The cell pellet was re-suspended in 10 mL 50 mM Tris–HCl buffer (pH 8.0). Lysozyme was added for a final concentration of 0.2 mg/mL and the mixture was incubated at 37 °C for 20 min and then sonicated. The supernatant was collected after a centrifugation (10,000 g for 10 min). The precipitate was resuspended in 10 mL 5% Triton X-100/50 mM Tris–HCl buffer (pH 8.0) and then the mixture was sonicated and incubated at 37 °C for 30 min. The supernatant was collected after a centrifugation (10,000 g for 10 min) and combined with the previous one for the subsequent Ni–NTA Superflow column chromatography (Qiagen, Venlo, the Netherlands). The bound protein was eluted using 0.3 M imidazole/0.15 M NaCl/50 mM Tris–HCl buffer (pH 8.0).

### Assay of the vanillin reduction activity of rCaCAD1 and the placental extracts by HPLC

To assay the vanillin reduction activity, 5 µg rCaCAD1 in a 100-µL reaction mixture and 35 µL CH-19 Sweet green fruit placental extract in a 50-µL reaction mixture were incubated with 4 mM vanillin and 1 mM NADPH (final concentrations) in 20 mM PKB (pH 6.8) at 30 °C. At each sampling time-point (from 1 to 24 h), a 10-µL aliquot of the reaction mixture was collected and added to 30 µL 0.2% trifluoroacetic acid (TFA) to terminate the reaction. The vanillyl alcohol and residual vanillin contents were measured using an HPLC system (Shimadzu Prominence LC-20A series; Shimadzu Co., Kyoto, Japan). A 5-µL aliquot of the terminated reaction mixture was loaded onto a Wakopak Fluofix-II 120E (4.6 × 250 mm) column (FUJIFILM Wako Pure Chemical Co., Osaka, Japan) equilibrated with 0.05% TFA at a flow rate of 1 mL/min. The elution involved a linear gradient of 5–10% methanol for the first 10 min and then a linear gradient of 10–60% methanol for the next 10 min. For the fluorescence detection, the excitation and emission wavelengths were 280 nm and 320 nm, respectively. The amount of synthesized vanillyl alcohol was estimated according to the peak area. The analysis of rCaCAD1 was also performed using a reaction solution lacking NADPH. The experiment using placental extracts was completed using samples from three different individuals.

### Reactions between rCaCAD1 and coniferyl aldehyde, cinnamaldehyde, or vanillin

Recombinant CaCAD1 (5 µg) and excess substrates (10 mM cinnamaldehyde, 1 mM coniferyl aldehyde, or 4 mM vanillin) were added to a 100-µL reaction mixture comprising 20 mM PKB (pH 6.8) and 1 mM NADPH. After a 30-min incubation at 30 °C, a 10-µL aliquot of the reaction mixture was collected and added to 30 µL 0.2% TFA to terminate the reaction. The products were analyzed by HPLC under the same conditions as those used for the vanillyl alcohol analysis.

### Heat deactivation of rCAD1 and placental extracts

Two sets of 5 µg rCaCAD1 and 35 µL CH-19 Sweet green fruit placental extract in 20 mM PKB (pH 6.8) were prepared. One set was incubated at 100 °C and the other set was incubated at 30 °C for 10 min. After the reaction mixtures cooled to room temperature, 4 mM vanillin and 1 mM NADPH (final concentrations) were added. Following a 30-min incubation at 30 °C, a 10-µL aliquot was collected and added to 30 µL 0.2% TFA to terminate the reaction. The products were analyzed by HPLC. The placental extract analysis was performed using samples from three different individual fruits.

### Synthesis of OHPAS

As previously described, OHPAS was synthesized via the condensation of 2-tert-butoxy-2-oxoethylzinc bromide with 2-hydroxy-*N*-sulfinylaniline^31^. The structure of the product was characterized by ^1^H NMR and fast atom bombardment mass spectroscopy.

### Inhibition of rCaCAD1 and placental extracts by OHPAS

Each reaction mixture (100 µL) contained 10 µg rCaCAD1 or 20 µL CH-19 Sweet green or red fruit placental extract, 4 mM vanillin, 1 mM NADPH, and 2, 2.5, 5, 10, or 20 mM (final concentrations) OHPAS in 20 mM PKB (pH 6.8). The reaction mixtures were incubated at 30 °C for 30 min or 1 h. At each sampling time-point, a 10-µL aliquot of each mixture was added to 30 µL 0.2% TFA to terminate the reaction. The amount of vanillyl alcohol synthesized was analyzed by HPLC as described above. The enzyme activity inhibition rate was estimated according to the peak area. The placental extract analysis was performed using samples from three different individual fruits.

### Inhibition of rCaCAD1 and placental extracts by EDTA

Each reaction mixture (100 µL) contained 5 µg rCaCAD1 or 40 µL CH-19 Sweet or red habanero green fruit placental extract, 4 mM vanillin, and 12.5, 25, 50, or 100 mM (final concentrations) EDTA in 20 mM PKB (pH 6.8). For the rCaCAD1 and CH-19 Sweet placental extract reaction mixtures, 1 mM NADPH was added as a proton donor. For the red habanero placental extract reaction mixtures, 16 mM gamma-amino butyric acid was added as an amine donor. The reaction mixtures were incubated at 30 °C for 30 min or 1 h. At each sampling time-point, a 10-µL aliquot of each mixture was added to 30 µL 0.2% TFA to terminate the reaction. The amount of vanillyl alcohol or vanillylamine synthesized was analyzed by HPLC as described above. The enzyme activity inhibition rate was estimated according to the peak area. The placental extract analysis was performed using samples from three different individuals.

### Syntenic analysis

The genomic synteny around the *C. annuum* genes *CaCAD1* (NM001324580), *pAMT* (LC423555), and *Pun1* (LC423556) was analyzed using the *C. annuum*, *S. tuberosum*, *S. lycopersicum*, *N. attenuata,* and *I. triloba* genomes in the Ensembl Plants (https://plants.ensembl.org/) and Genomicus (https://www.genomicus.bio.ens.psl.eu/genomicus-plants-49.01/cgi-bin/search.pl) databases. The TBLASTN algorithm was used to screen the genome sequences of other species for sequences similar to the *C. annuum* CAD1, pAMT, and Pun1 sequences. Query coverage greater than 80% and sequence homology exceeding 70% were set as the criteria for identifying CAD, pAMT, and Pun1 homologs for the synteny analysis.

### Ethical statement

The experimental methods were approved by the Ethical Committee of Josai University, and performed in strict conformance with relevant guidelines and regulations.

## Supplementary Information

Supplementary Information.

## Data Availability

The data that support the findings of this study are available from the corresponding author, KS, upon reasonable request. *Capsicum annuum* CAD (CaCAD1): NM001324580, *Capsicum annuum* pAMT: LC423555, *Capsicum annuum* Pun1: LC423556, *Capsicum annuum* β-actin: AY572427, *Populus trichocarpa* CAD: EU603306, *Nicotiana tabacum* CAD (NtCAD): X62344, *Arabidopsis thaliana* CAD (AtCAD5): NM_119587.
