# Supplementary material for: Vanillin reduction in the biosynthetic pathway of capsiate, a non-pungent component of Capsicum fruits, is catalyzed by cinnamyl alcohol dehydrogenase
Source: Sci Rep. 2022 Jul 20;12:12384. doi: 10.1038/s41598-022-16150-1 (PMC9300701; doi:10.1038/s41598-022-16150-1)
Supplement: Supplementary file 1 — Supplementary Information. [file 41598_2022_16150_MOESM1_ESM.pdf]

Supplementary Information

Vanillin reduction in the biosynthetic pathway of capsiate, a non-pungent component of *Capsicum* fruits, is catalyzed by cinnamyl alcohol dehydrogenase

Kaori Sano*[1], Yuya Uzawa[1], Itsuki Kaneshima[2], Saika Nakasato[2], Masashi Hashimoto[1], Yoshiyuki Tanaka[3], Sachie Nakatani[2], and Kenji Kobata*[2].

1. Department of Chemistry, Faculty of Science, Josai University, Saitama, Japan

2. Graduate School of Pharmaceutical Sciences, Josai University, Saitama, Japan

3. Graduate School of Agriculture, Kyoto University, Kyoto, Japan

*Corresponding authors

Kaori Sano

Department of Chemistry, Faculty of Science, Josai University, 1-1, Keyakidai, Sakado, Saitama, Japan

Tel: +81-49-271-7687

E-mail: kaori-s@josai.ac.jp

Kenji Kobata

Graduate School of Pharmaceutical Sciences, Josai University, 1-1, Keyakidai, Sakado, Saitama, Japan

Tel: +81-49-271-7654

E-mail: kobata@josai.ac.jp

The supplementary information includes four Additional Figures, four Tables and one Reference.

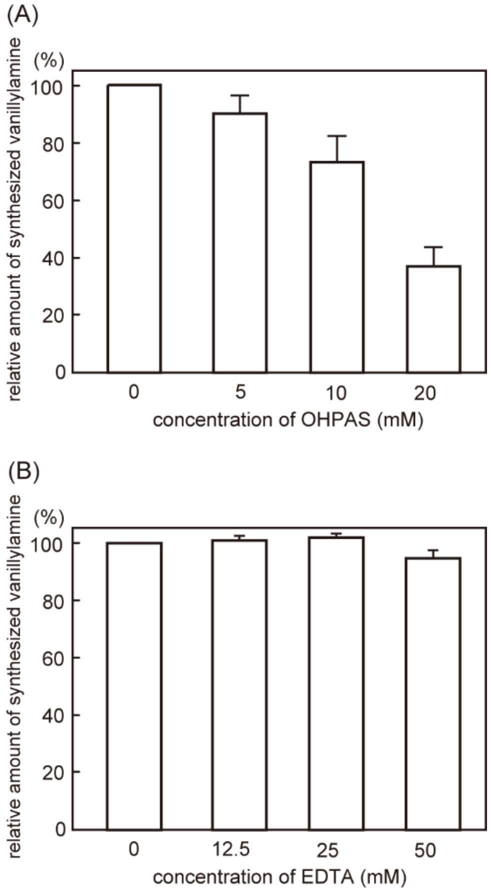

Figure S1. Inhibition of the vanillylamine synthesis reaction in placental extracts of red habanero by inhibitors, OHPAS and EDTA.

Vanillylamine synthesis activity of placental extracts of immature green fruits was measured in the presence of (A) 0–20 mM OHPAS and (B) 0–100 mM EDTA. Amount of vanillylamine synthesized in the absence of inhibitors was set as 100% and the amount synthesized relative to it is shown. Individual three fruits were used for all experiments (*n=3*).

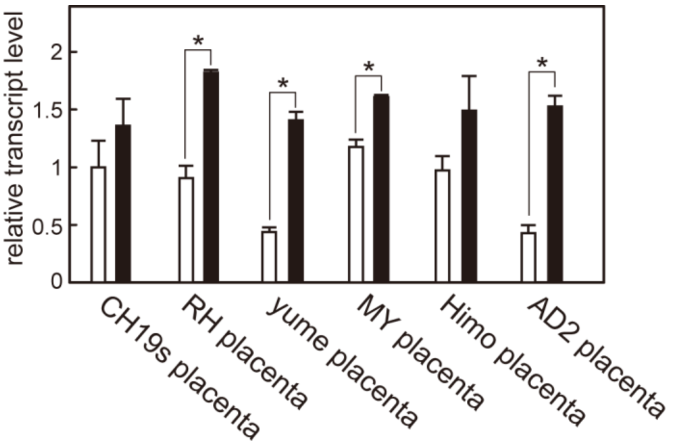

Figure S2. Quantitative PCR analyses of *CAD*

Transcript levels of CAD in placenta of CH-19 sweet (CH19s), red habanero (RH), Yumematsuri (yume), Morga Yellow (MY), Himo and (AD2) were compared. Expression was normalized against that of average of three control genes. The relative transcript level of each gene was calculated using the comparative Ct method, with the expression level of each gene in CH-19 sweet set to 1. White and black bars indicate immature green and mature red fruits, respectively. Asterisks indicate $P < 0.05$: significance by the Welch t-test. Each experiment was performed using RNA extracted from three different individual fruits (n=3).

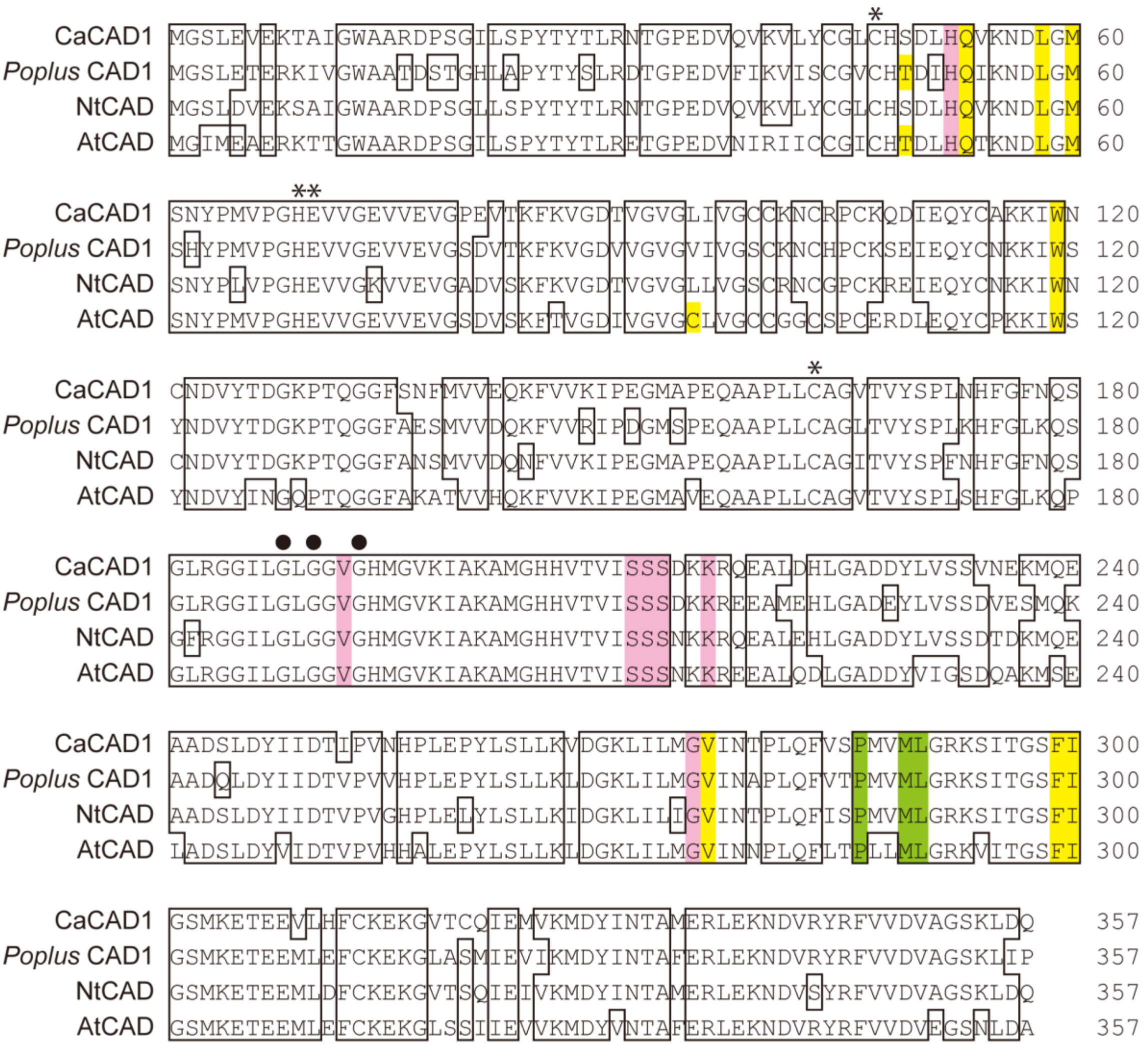

Figure S3. Alignment of CADs.

Amino acid sequences of CADs were compared between CaCAD1 (cloned from CH-19 sweet in the present study, CaCAD1), *Populus trichocarpa* CAD (CAD1, EU603306), *Nicotiana tabacum* CAD (NtCAD, X62344), and *Arabidopsis thaliana* CAD (AtCAD5, NM_119587). Eukaryotic CAD works with dimers. The conserved residues constituting a substrate-binding site are highlighted by yellow and green colors indicating their belonging to different subunits. The amino acids interacted with catalytic Zn^{2+} ion is marked with asterisk (Cys47, His69, Glu70 and Cys163). The amino acids held $NADP^+$ molecule is indicated in pink box. The conserved glycine residues in GX(X)GXXG motif are marked with dots (1).

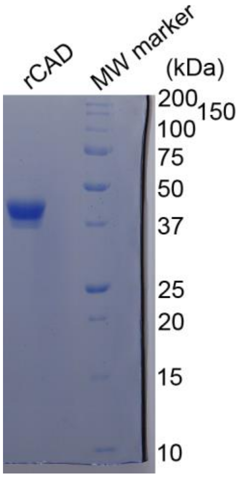

Figure S4. SDS-PAGE pattern of rCAD.

Purified rCAD were analyzed by SDS-PAGE. The numbers on the right refer to the size of molecular mass makers.

Table S1 Vanillyl alcohol synthesis reaction of extracts treated by heating

To clarify whether heating suppress the vanillyl alcohol synthesis reaction, the placental extracts of green fruit of CH-19 sweet was heated at 100°C and then reacted with vanillin. (n=3)

pretreatment	no	100°C
VOH (nmol)	2.8	ND

Table S2 Vanillyl alcohol synthesis reaction of rCaCAD1

Before reaction with vanillin, rCaCAD1 was pre-treated at 30°C or 100°C for 10min. As another experiment, the reaction with and without NADPH was also performed.

pretreatment	30°C	30°C	100°C
NADPH	with	without	with
VOH (nmol)	6.4	ND	ND

Table S3. Primer sets for real-time PCR.

**CAD**	Forward: GGGTGGCTTTTCTAATTTCATGGTTG
	Reverse: GCCTTTGCTATTTTCACTCCCATGT
**pAMT**	Forward: CCACTTACATTTCTGCTGGTCTCTC
	Reverse: CAATGAAAGCAGCTACTGTTTCAGG
**Pun1**	Forward: GCCTTGGGCGAATAATTGTGAAG
	Reverse: TTAAGCAGAGAGCAACCATCACC
**β-actin**	Forward: AGCAACTGGGACGATATGGAGAAG
	Reverse: AAGAGACAACACCGCCTGAATAGC
**UBQ**	Forward: GCACAAGCACAAGAAGGTTAAG
	Reverse: GCACCACACTCAGCATTAGGA
**EF1α**	Forward: AAGATCGACAGGCGTTCAGGTAAG
	Reverse: TGGTGGGTATTCAGCAAAGGTTTC

Table S4. Primer sets for cloning of CAD cDNA of CH-19 sweet and habanero.

Forward: ATGGGTAGCTTGGAAGTTGAGAAAACAGC

Reverse: TTACTGGTCAAGTTTGCTTCCAGCAACATC

Reference

1. B. Youn, R. Camacho, S. G. A. Moinuddin, C. Lee, L. B Davin, N. G Lewis, C. Kang, Crystal structures and catalytic mechanism of the Arabidopsis cinnamyl alcohol dehydrogenases AtCAD5 and AtCAD4. *Org. Biomol. Chem*. **4**, 1687-1697 (2006).
